# Metataxonomic and Metagenomic Approaches vs. Culture-Based Techniques for Clinical Pathology

**DOI:** 10.3389/fmicb.2016.00484

**Published:** 2016-04-07

**Authors:** Sarah K. Hilton, Eduardo Castro-Nallar, Marcos Pérez-Losada, Ian Toma, Timothy A. McCaffrey, Eric P. Hoffman, Marc O. Siegel, Gary L. Simon, W. Evan Johnson, Keith A. Crandall

**Affiliations:** ^1^Computational Biology Institute, The George Washington UniversityAshburn, VA, USA; ^2^Facultad de Ciencias Biológicas, Center for Bioinformatics and Integrative Biology, Universidad Andres BelloSantiago, Chile; ^3^Centro de Investigação em Biodiversidade e Recursos Genéticos (CIBIO-InBIO)Vairão, Portugal; ^4^Children's National Medical Research CenterWashington DC, USA; ^5^Division of Genomic Medicine, Department of Medicine, The George Washington University School of Medicine and Health SciencesWashington DC, USA; ^6^Division of Genomic Medicine, Department of Medicine, Department of Microbiology, Immunology, and Tropical Medicine, The George Washington University School of Medicine and Health SciencesWashington DC, USA; ^7^Division of Infectious Diseases, Department of Medicine, School of Medicine and Health Sciences, The George Washington UniversityWashington DC, USA; ^8^Computational Biomedicine, Boston University School of MedicineBoston, MA, USA

**Keywords:** microbiome, metagenomics, metataxonomics, high throughput sequencing, drug resistance, pathogen detection

## Abstract

Diagnoses that are both timely and accurate are critically important for patients with life-threatening or drug resistant infections. Technological improvements in High-Throughput Sequencing (HTS) have led to its use in pathogen detection and its application in clinical diagnoses of infectious diseases. The present study compares two HTS methods, 16S rRNA marker gene sequencing (metataxonomics) and whole metagenomic shotgun sequencing (metagenomics), in their respective abilities to match the same diagnosis as traditional culture methods (culture inference) for patients with ventilator associated pneumonia (VAP). The metagenomic analysis was able to produce the same diagnosis as culture methods at the species-level for five of the six samples, while the metataxonomic analysis was only able to produce results with the same species-level identification as culture for two of the six samples. These results indicate that metagenomic analyses have the accuracy needed for a clinical diagnostic tool, but full integration in diagnostic protocols is contingent on technological improvements to decrease turnaround time and lower costs.

## Introduction

Infectious diseases remain a significant health care burden in both the United States and worldwide. In 2011, infectious diseases accounted for ~4.0 million emergency department visits (Ambulatory and Hospital Care Statistics, 2016)1^1^, 3.9 million outpatient department visits (Ambulatory and Hospital Care Statistics, 2016)1 and ~4% of patients in acute care hospitals develop a health care associated infection (Magill et al., 2014). The accurate identification of the pathogen(s) causing disease is crucial to the correct diagnosis and treatment for the infection. A comprehensive, accurate, and rapid diagnosis, including pathogen identification at the species level and antibiotic resistance pattern, enables physicians to use more targeted antimicrobial therapies for these patients (Didelot et al., 2012). Currently, there are many methods that can be used to characterize the microbial composition of a sample from an infected patient and identify the potentially causative agent(s) including culture, polymerase chain reaction (PCR), microarrays, and High-Throughput Sequencing (HTS) (Table 1).

**Table 1:** **Taxonomic identification methods comparisons**.

**Technique**	**Speed (days)**	**Cost**	**Accuracy**	**Resistance mutations**	**Multispecies**	**High-**	**Emergent**	**Reference**
						**throughput**	**pathogens**	**update**
Culture	2–14	$	Genus or Species	No, additional assays	No	No	No	No
PCR	2	$	Genus or Species	No, additional assays	No, additional assays	No	No	No
Microarray	2	$	Genus or Species	Yes	Yes	Yes	No	Annually but costly
16S	1.5–2	$$	Genus or Species	No	Yes	Yes	Yes	Every run
Metagenomic	2–3	$$	Species or Strain	Yes	Yes	Yes	Yes	Every run

Microbial culture has been considered the gold standard of diagnostic techniques for bacterial and fungal species and is widely used in clinical laboratories. Culture methods involve growing the pathogen on appropriate media, a method developed more than 100 years ago (Didelot et al., 2012). Further identification of the pathogen, especially to the species level, often requires biochemical tests. Additional tests, such as antibiotic resistance tests, may require additional cultures. Due to the limitations of the media utilized for growth, there will be an inherent bias to cultures. Culture methods can only confirm the presence of a microorganism that can grow on the selected media. Therefore, culture techniques may not be effective at identifying the presence of novel pathogens or known but unculturable pathogens (e.g., environmental and clinical isolates; Didelot et al., 2012). Nevertheless, culture remains the most widely used diagnostic method based on its extensive validation and cost-effectiveness (Köser et al., 2012).

Traditional PCR and, more recently, microarray assays analyze a pathogen's genetic profile, rather than the morphological, phenotypic, and biochemical features that standard culture methods utilize. PCR assays are quick, specific, and cheap when the list of possible targets is short. PCR's weakness as a diagnostic tool stems from its potential bias, because target sequences for primer design must be chosen before testing begins. Further, PCR has relatively low-throughput capabilities, because multiple PCR amplifications per sample negatively impact the cost- and time effectiveness of the method (Wang et al., 2006; McLoughlin, 2011).

High-density microarrays are able to detect thousands of pathogens simultaneously through the design of specific, degenerate, and tiled oligonucleotide probes. However, these probes need to be periodically updated as new genomes are sequenced, which is a time consuming and expensive process. Universal microarrays have been attempted, but exhibit bias as probe or target secondary structure can influence hybridization in an unpredictable pattern (Yang and Rothman, 2004; McLoughlin, 2011). PCR and microarrays are both able to leverage genetic differences in pathogens for greater resolution than culture methods, but the inherent bias found in both PCR and microarrays still presents an obstacle to achieve an efficient and agnostic diagnostic tool.

HTS is the most in-depth and unbiased method of obtaining genomic or metagenomic information (Metzker, 2009). Unlike PCR or microarrays, it does not require primer or probe design, it can be easily multiplexed, and the specificity and selectivity of the sequencing can be adjusted computationally after acquiring the data (Adams et al., 2009; Dunne et al., 2012). The two main methods of pathogen identification using HTS are marker sequencing, chiefly 16S ribosomal RNA (16S) but also internal transcribed spacer (ITS) region for fungal species, and whole metagenomic shotgun sequencing. We adopt the nomenclatural suggestions of Marchesi and Ravel (Marchesi and Ravel, 2015) and refer to the high throughput marker (e.g., 16S or ITS) based approach to microbial diversity characterization as *metataxonomics* and the shotgun genomic sequencing approach as *metagenomics*. A common weakness for HTS methods is database bias, but as sequencing becomes cheaper and more widely used, databases are growing in size and diversity. For example, in 2014, the FDA GenomeTrakr project uploaded to NCBI an average of 848 *Salmonella* and *Listeria* genomes per month (Allard et al., 2016).

Metataxonomics using 16S sequencing is a widely used technique that relies on the conserved and variable regions of the bacterial 16S rRNA gene to make community-wide taxonomic classifications. As HTS technologies have improved, the read length and overall quality of the sequencing has also improved, allowing for greater species resolution (Klindworth et al., 2012). 16S sequencing is the most widely used technique for microbial diversity analysis and has been used to investigate various environments, from soil in Antarctica (Chong et al., 2012) to the human gut (Dethlefsen et al., 2008). Because the 16S HTS approach is a PCR-based approach, it suffers from the same issues described above for PCR. 16S sequencing only uses data from one multicopy gene, any two organisms with the same 16S rRNA gene sequence might be classified as the same strain under a 16S analysis, even if they were, in reality, different strains. For example, based on 16S sequences, *Escherichia coli O157:H7* cannot be differentiated from *E. coli K-12* (Weinstock, 2012) nor *Shigella flexneri* from *E. coli*. The 16S gene has been shown to have intra-organismal differences, such as multiple copies (Rajendhran and Gunasekaran, 2011) and intra-genomic heterogeneity (Rajendhran and Gunasekaran, 2011), which will negatively influence the method's accuracy.

Metagenomic sequencing avoids PCR bias and it is not restricted to only bacterial sequences. In addition, the coverage of the genome outside of the small 16S rRNA gene region means that specific, strain level discrimination is achievable. This has been shown in the metagenomic sequencing of cholera (Chin et al., 2011), tuberculosis (Gardy et al., 2011), *E. coli* (Rasko et al., 2011), and methicillin-resistant *Staphylococcus aureus* (MRSA) (Köser et al., 2012). Currently, the high cost of metagenomic sequencing and the noisy signal due to host contamination are the greatest drawbacks of this approach. Metagenomic sequencing captures not only the pathogenic sequences, but also the human host's genetic material, which can overwhelm the signal from the pathogens (Kuczynski et al., 2011) and lead to an inaccurate classification of the pathogenic community. On the other hand, the human host genetic sequences can be an advantage in examining a genetic response to infection (Perez-Losada et al., 2015). However, metagenomic sequencing is also much more expensive than 16S sequencing, especially to achieve the coverage and depth needed for species identification (Quail et al., 2012).

Metataxonomics and metagenomics, with their culture independence and wealth of data, both have the potential to improve diagnostics. But before either one of these methods can become fully integrated into diagnostic protocols, their relative benefits need to be compared and validated by culture methods (Dunne et al., 2012). In this study, 16S and genomic DNA shotgun sequencing are compared in their respective abilities to match the clinical, culture diagnosis of intubated patients being clinically assessed for possible ventilator associated pneumonia (VAP). Patients with VAP have a crude mortality rate of 20–70% and an attributable mortality rate of 10–40% (Heyland et al., 1999; Ashraf and Ostrosky-Zeichner, 2012; Luyt et al., 2013). A faster and more accurate pathogen diagnosis would hopefully lead to targeted antimicrobial therapy, thereby reducing the excessive use of broad-spectrum antibiotics, their antibiotic-associated side effects and healthcare costs (Aryee and Price, 2014) and possibly the mortality rate in these patients (Dupont et al., 2001).

## Materials and methods

In order to evaluate the ability of HTS to match the culture inference, bronchial aspirate samples were taken from eight intubated patients from The George Washington University Hospital with suspected VAP. Three methods—16S sampling, metagenomic sampling, and traditional culturing—were employed to determine the infectious agent (Table 2). The collection of discarded aspirate samples for bacterial sequencing and de-identified clinical and microbiological data was approved by the GWU Institutional Review Board. The indication for endotracheal suctioning was solely based on the clinical evaluation of the patient's attending physician, as was the decision regarding any antibiotic therapy.

**Table 2:** **Culture, metagenomic, and metataxonomic (16S) inferences across samples**.

**Sample code**	**Metagenomic accession numbers**	**Culture inference**	**HTS inference**					
			**Metagenomic**			**Metataxonomic (16S)**		
			**Strain**	**ti number**	**Abundance**	**Strain**	**Silva ID number**	**Abundance**
s002	SRX682947	Moderate *Acinetobacter baumanii*	*Acinetobacter baumannii* TCDC-AB0715	980514	0.333328772	*Stenotophomonas maltophilia*	AB294553	0.831492105
			*Delftia acidovorans* SPH-1	398578	0.097767298	*Acinetobacter baumannii*	X81660	0.166800495
			*Escherichia coli* str. K-12 substr. MG1655	511145	0.071375325	*Achromobacter denitrificans*	AJ278451	0.001707401
			*Acidovorax* sp. JS42	232721	0.057764467			
			Uncultured bacterium	77133	0.041203617			
s014	SRX682948	Moderate yeast	Human herpesvirus 1	10298	0.748475222	*Stenotrophomonas maltophilia*	AB294553	0.996154442
			*Delftia acidovorans* SPH-1	398578	0.048150122	*Phocaeicola abscessus*	EU694176	0.002876914
			*Escherichia coli* str. K-12 substr. MG1655	511145	0.036559297	*Achromobacter denitrificans*	AJ278451	0.000955617
			*Candida parapsilosis*	5480	0.020277269			
			Uncultured bacterium	77133	0.017402208			
s017	SRX682949	Moderate *Klebsiella*, moderate yeast	*Candida glabrata*	5478	0.300566414	N/A		
			*Streptococcus pasteurianus* ATCC 43144	981540	0.219646355			
			*Klebsiella pneumoniae*	573	0.087924829			
			*Delftia acidovorans SPH-1*	398578	0.074917391			
			*Achromobacter xylosoxidans* A8	762376	0.041784424			
s043	SRX682950	Moderate *Pseudomonas aeruginosa*	*Pseudomonas aeruginosa* PAO1	208964	0.764117144	*Pseudomonas otitidis*	AY953147	0.945600683
			*Pseudomonas aeruginosa*	287	0.059077537	*Gemella haemolysans*	L14326	0.03173556
			*Pseudomonas aeruginosa* DK2	1093787	0.054320167	*Staphylococcus aureus*	L36472	0.020936643
			*Acinetobacter baumannii* TCDC-AB0715	980514	0.022756707			
			*Pseudomonas phage* DMS3	389469	0.021813898			
s049	SRX682951	Moderate *Pseudomonas aeruginosa*	*Pseudomonas aeruginosa* PAO1	208964	0.805335723	*Pseudomonas otitidis*	AY953147	0.954942809
			*Pseudomonas aeruginosa*	287	0.132182547	*Azomonas agilis*	AB175652	0.037916596
			*Delftia acidovorans* SPH-1	398578	0.015413769	*Acinetobacter baumannii*	X81660	0.007140139
			*Pseudomonas aeruginosa* LESB58	557722	0.005846147			
			*Pseudomonas aeruginosa* PA7	381754	0.005035477			
s070	SRX682952	Abundant oropharyngeal flora	*Neisseria meningitidis* MC58	122586	0.139880136	*Neisseria cinerea ATCC 14685*	ACDY02000019	0.964024816
			Uncultured bacterium	77133	0.122063293	*Streptococcus mitis*	AF003929	0.018385596
			*Candida glabrata*	5478	0.071585191	*Corynebacterium pseudodiphtheriticum*	AJ439343	0.015650335
			*Streptococcus pasteurianus* ATCC 43144	981540	0.070621397			
			*Neisseria meningitidis* Z2491	122587	0.067880228			
s071	SRX682953	Abundant MRSA	*Staphylococcus aureus* subsp. aureus JH1	359787	0.877861749	*Staphylococcus aureus*	L36472	0.997915382
			*Staphylococcus epidermidis*	1282	0.04124046	*Enterococcus faecalis*	AB012212	0.002038779
			*Enterococcus faecalis V583*	226185	0.014553905	*Raoultella ornithinolytica*	U78182	0.0000427
			*Staphylococcus epidermidis* ATCC 12228	176280	0.011848339			
			*Candida glabrata*	5478	0.010538713			
s074	SRX682954	Moderate GPCs, moderate *Streptococcus pneumoniae*	*Candida glabrata*	5478	0.453943438	N/A		
			Uncultured fungus	175245	0.193800314			
			*Streptococcus pasteurianus* ATCC 43144	981540	0.079202375			
			*Pseudomonas aeruginosa* LESB58	557722	0.044692608			
			*Delftia acidovorans* SPH-1	398578	0.036024421			

### DNA preparation for HTS

We followed the DNA extraction protocol of Toma et al. (2014). Illumina's Nextera DNA Sample Preparation Kit was used to generate and barcode the sequencing libraries for the genomic DNA (gDNA). The gDNA was sequenced on a HiSeq 2500 (100 bp; single-end reads; NCBI accession number SRP045601). Six of the eight samples were sequenced twice and the sequence results were combined into one fastq file. Six of the samples corresponded to the ones described in Toma et al. (2014) and full-length PCR-amplified 16S sequences using PacBio sequencing were obtained from NCBI under accession numbers SRP028704 and SRP031650 (Toma et al., 2014). We acknowledge the sample size is limited, yet these were the only samples available for follow up DNA work.

### Culture

The deep endotracheal aspirates were submitted to the GWU Hospital microbiology laboratory for routine Gram-staining and microbial culture as described (Toma et al., 2014). In short, the most purulent or blood-tinged portions were used for a Gram-stain and bacterial culture on sheep blood, chocolate and MacConkey agars. The cultures on sheep blood and chocolate agars were incubated in 5% CO_2_ at 35°C for at least 48 h, while the cultures on MacConkey agar were incubated in a non-CO_2_ atmosphere at 35°C for at least 24 h. Significant growth was defined as moderate to heavy growth of an isolate in the second, third, or fourth quadrants of each plate. Organisms identification and susceptibility results were accomplished using the Vitek® 2 identification (ID) and antibiotic susceptibility testing (AST) cards (bioMériuex Marcy l'Etoile, France) following the standard operating procedures utilized by the GWU Hospital microbiology laboratory. The residual aspirate samples were frozen at −80°C until processing for DNA extraction. We used the culture results as the “gold standard” against which we compared the metataxonomic and metagenomic results.

### Quality control for HTS data

The raw metagenomic reads were preprocessed using PrinSeq-Lite v. 0.20.3 (filtering reads and trimming 3′ and 5′ bases < 25 PHRED, removing exact duplicates, reads with undetermined bases, and low complexity reads using Dust filter = 30) (Schmieder and Edwards, 2011). Human reads were filtered using Bowtie2 (Langmead and Salzberg, 2012) by mapping the metagenomic reads against the latest human genome reference (hg19) (Church et al., 2011). The raw 16S reads were processed through the PacBio SmartPortal pipeline to filter out reads shorter than 100 bp, reads with no insert, low complexity or low quality reads and to trim adaptor sequences as described in Toma et al. (2014).

### Taxonomic profiling with 16S and metagenomic data

We used PathoLib from PathoScope 2.0 (Hong et al., 2014) to obtain all the sequence data under the bacterial, viral, and fungal taxonomy IDs from NCBI's nucleotide non-redundant (NCBI nt nr) database as of March 3, 2014 and downloaded “The All-Species Living Tree” Project (LTP) (Yarza et al., 2008; Munoz et al., 2011) 16S database, version 113. We mapped the metagenomic reads against the bacterial, viral, and fungal NCBI nt nr database and then against the hg19 and phix174 database using the PathoScope 2.0 PathoMap module. The reads that mapped with a higher score to the hg19 or the phix174 database were removed. Similarly, we mapped the 16S sample reads against the LTP database and then against the hg19 and phix174 databases, removing the reads that mapped with a higher score to the hg19 or the phix174. We applied a Bayesian mixture read reassignment model (Francis et al., 2013), as implemented in the PathoID module of PathoScope 2.0, to both of the PathoMap mapping results. If a sample's results had multiple strains of the same species in the top hits, the PathoID module was run again with a new theta parameter of 10,000. In order to determine the presence or absence of the *mecA* gene (GenBank Acession number NX52593), we used BLAST+ (Camacho et al., 2009) to create a blast database from the reads of each sample's metagenomic sequences and queried the *mecA* gene sequence against each database using BLAST. All analyses were conducted on GWU's ColonialOne High-Performance computer cluster and computational time was recorded using the linux time(1) module.

### Lab validation of fungal DNA

To confirm the presence of fungal DNA detected by the metagenomic analysis, we performed subsequent PCR using fungal ITS primers to verify inferred taxa from the original samples for which there was no 16S amplification. A PCR reaction for sequencing was performed using universal primers ITS1F and ITS4 targeting the nuclear ITS region (White et al., 1990) in samples s017 and s074. Amplification was performed in a 15 μL reaction volume consisting of 10.025 μL nuclease free water, 12.5 μL GoTaq green buffer (Promega, Madison, WI), 0.3 μL forward primer, 0.3 μL reverse primer, 0.3 μL dNTPs, 0.075 μL GoTaq (Promega, Madison, WI), and 1 μL of template, either gDNA from sample s017 or s074. PCR was performed using a Mastercycle Nexus Gradient PCR machine (Eppendorf). The thermal cycler was programmed for 2 min at 95°C for initial denaturation, followed by 30 cycles of 1 min at 95°C for denaturation, 1 min at 52°C for annealing, 3 min at 72°C for extension, and 10 min at 72°C for the final extension.

We cleaned the PCR products using ExoSAP (Affymetrix, Santa Clara, CA). Each ExoSAP reaction contained 1 μL PCR product and 2 μL of ExoSAP diluted 1:3, and the thermal cycler was programed for 37°C for 15 min and 80°C for 15 min. Each product was sequenced in both directions and in duplicates. The cycle sequencing reaction contained 6.75 μL water, 0.5 μL primer (forward or reverse), 1.25 μL 5X buffer, 0.5 μL Big Dye (Life Technologies) mix, and 1 μL of the cleaned PCR product. The thermo cycler was programed for 30 cycles of 95°C for 30 s, 50°C for 30 s, and 60°C for 4 min. ITS amplicons were sequenced on an ABI 3730XL platform.

We revised chromatograms, adjusted quality, and created the consensus sequences, one contig for sample s017 and two contigs for sample s074, in Geneious v. 7.1.5 (Kearse et al., 2012). These consensus sequences were aligned along with 32 other sequences from *Candida glabrata, C. albicans, C. dubliniensis, C. parapsilosis, C. rugosa, C. tropicalis*, and *Malassezia globosa* using MUSCLE alignment with 8 iterations (Supplementary Table 1). These species were chosen either because they were identified by the metagenomic analysis (*C. glabrata, C. tropicalis, C. albicans*, and *M. globosa*) or because they are closely related to the top hit, *C. glabrata*, (*C. dubliniensis* and *C. rugosa*). A maximum-likelihood tree was created using RAxML (Stamatakis, 2006) with 50 independent replicates and 10,000 bootstrap replicates. Analyses were performed using Geneious v. 7.1.5 (Kearse et al., 2012) and SumTrees (Guss et al., 2011).

## Results and discussion

Our study resulted in three data sets. First, the 16S data (PacBio) which was collected previously (Toma et al., 2014) and raw data files with sequence from each patient were submitted in bas.h5 format to the NCBI Short Read Archive (SRA) under the accession numbers SRP028704 and SRP031650. Second, metagenomic data are new to this study and sequences were submitted in fastq format to NCBI SRA under the accession numbers SRX682947 to SRX682954. Third, the fungal ITS data are new to this study and those amplicon target sequences were deposited at NCBI under accession numbers KU936092-KU936095. Using these data, we found differences in the abilities of the metataxonomic approach vs. the metagenomic approach to identify pathogens characterized through culture techniques. Computational time also differed between approaches. Furthermore, the metagenomic approach identified fungal pathogens not captured in the metataxonomic approach or culture approach. These comparisons are detailed below.

### The effect of sequencing type on computational time

The mapping stage for the 16S analysis took an average of 7 min and 13 s and used an average of 50.65 CPU seconds in kernel. The mapping stage of the metagenomic analysis took an average of 23 h and 1 min and used an average of 80,427.72 CPU seconds in kernel, ~1500 times longer.

As predicted, the 16S analysis was much faster than the metagenomic analysis, due to the smaller number of sequences and the smaller database (Supplementary Table 2). The average 16S analysis took 5% of the time of the average metagenomic analysis and used only 0.1% of the CPU seconds. The turnaround time for each method is important because the integration of HTS into diagnostics hinges in part on whether or not a diagnosis can be produced as fast as or faster than current culture methods. A timely diagnosis allows clinicians to prescribe treatments quickly, which should improve the outcome of the treatment. Culture methods take on average 48 h to produce results (Didelot et al., 2012; Köser et al., 2012). Based on our results, the 16S analysis, at its most efficient, can match the 48 h turnaround timeline of culture, but the metagenomic analysis currently takes longer than the culture methods. The cost-effectiveness, efficiency, and coverage of metagenomic sequencing, and HTS in general, varies among different machines (Quail et al., 2012). However, if current trends continue, higher coverage with a shorter run time and lower cost should be achievable as the technology improves. While time and cost will not affect downstream analyses, an increase in coverage will increase the already time-intensive mapping stage. Before a metagenomic analysis can be considered comparable to culture methods, improvements will have to be made in the mapping stage of the taxonomic profiling to decrease the amount of time required for metagenomic sequencing. It should be noted that the 48-h turnaround for culture methods is not true for all bacteria. Slow-growing bacteria or bacteria that require a series of cultures for diagnosis could take upwards of 2 weeks to complete the identification (Didelot et al., 2012). Likewise, fungal culture growth can take up to 1 week. Metagenomic sequencing does not depend on a culture step and all of the genetic information should, in theory, be collected through DNA extraction of the primary sample in a single step. Consequently, metataxonomic and metagenomic analyses are not affected by slow-growing or multi-culture pathogens and the turnaround times for these analyses have much smaller ranges than for the culture methods (Didelot et al., 2012).

### Taxonomic identification

The metataxonomic (16S) analysis identified an average of 8.5 operational taxonomic units (OTUs) per sample with a range of 3-18 OTUs. We designate OTUs as 16S (as mentioned in the introduction) often does not have resolving power for species and certainly strains due to the lack of sequence divergence at this one conserved locus (Caro-Quintero and Ochman, 2015). However, our approach does capitalize on the PacBio platform to achieve full-length amplimers of the 16S (~1500 bps) with greater than 100x coverage resulting in very high accuracy compared to many metataxonomic approaches that use short-read technology with lower coverage (Toma et al., 2014). The metagenomic analysis identified a larger number of OTUs with an average of ~374 OTUs per sample with a range of 185-797 OTUs. This high level of diversity is not unexpected; studies of the lung microbiome have found over 50 different bacterial genera in the lungs (Erb-Downward et al., 2011; Guss et al., 2011). For four of the samples, the metagenomic analysis identified several OTUs of the top hit species, while the 16S analysis did not contain any strain differentiation due to the lack of resolving power of this single locus approach. Multiple OTUs in the top hits could be indicative of multiple strains of the same species circulating in the sample or the presence of a novel species not found in the database. Samples s043 and s049 both had multiple OTUs in their top hits and the possibility of each scenario described above was explored using the PathoID theta parameter. The theta parameter controls the assumed number of ambiguous reads in a sample. Raising the theta parameter to 10,000 from the default assumes a more even distribution over several genomes in the database. A lack of change in the overall distribution between the results with the increased theta parameter vs. the default parameter indicates the top hit identified by PathoScope is most likely the correct strain in each sample (Supplementary Table 3).

**Figure 1:**
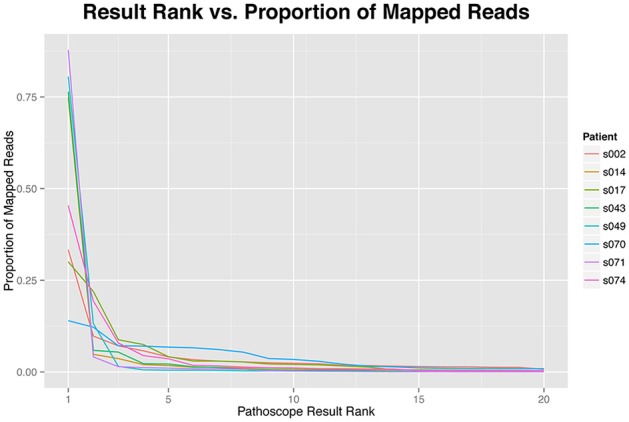
**The proportion of reads mapping to each identified taxonomic unit by results rank**. The proportion of reads are highly skewed toward the top hits, more than 75% of the reads of each sample are represented by the first five results.

While the increased diversity of hits in HTS analysis compared to PCR or microarrays may provide additional information, it has been shown that PathoScope and other Bayesian Mixture Models might create a tail of false positives (Lindner and Renard, 2015; Morfopoulou and Plagnol, 2015) (Figure 1). In the context of a clinical diagnosis, this tail of false positives should not influence the final interpretation. We assume any pathogen causing an infection would be expected to constitute a large percentage of the pathogen population present in a sample and therefore would not be included in the tail of false positives. There are diseases for which this assumption will not hold, that is, the pathogen will not be the microbe in greatest abundance. This is especially true in non-sterile settings such as the gut, lung, nasal passage, where there is an abundance of microbes, some commensal, some opportunistic, and perhaps some pathogenic. Under these circumstances, it is essential to have appropriate controls for direct comparison of potentially pathogenic microbes that differ from “normal” microbiome components. With the metagenomic approach with RNAseq reads, one can also use the host data to examine host response to potential “infection” to help validate the identification of pathogens (Castro-Nallar et al., 2015).

### The effect of sequencing on clinical diagnosis

**Figure 2:**
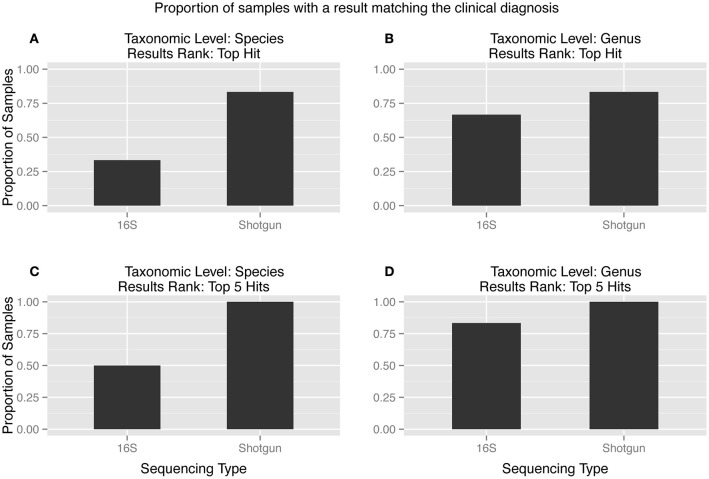
**(A–D):** Proportion of samples matching the clinical diagnosis at different taxonomic levels and results rank. The metagenomic analysis is more accurate than the 16S analysis in every scenario. The ideal combination, a match between the clinical diagnosis and the tophit at the species level, showed the largest discrepancy between the two sequencing types.

The top hit from the metagenomic analysis was consistent with the culture inference at the species level in five of the six samples, while the culture inference and the 16S analysis top hit matched at the species level for only two of the six samples (Figure 2A). The two sequencing methods were only close in accuracy when the matches were considered at the genus level and the top five hits were taken into account (Figure 2D). The two samples that did not produce 16S amplification, s017 and s074, both had top hits of *C. glabrata*. This diagnosis is consistent with culture inference of s017 but not s074.

Sample s014 was the only sample that did not have the clinical diagnosis, i.e., yeast, matched as the top hit by either HTS method. However, the metagenomic analysis reported the fungus *C. parapsilosis* as the fourth hit with 2% of the reads. The 16S analysis never identified yeast, which is expected because the LTP database only contained bacterial sequences and 16S sequencing is restricted to bacteria. This is the only sample of the six samples analyzed by both HTS methods for which the metagenomic analysis did not recover an organism that was identified by culture techniques.

**Figure 3:**
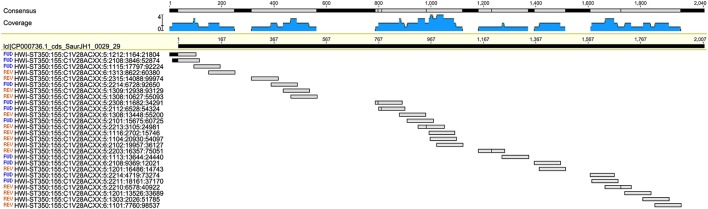
**Twenty-seven metagenomic reads from sample s070 mapping to the ***mec-A*** (penicillin-binding protein CDS) region of the ***Staphylococcus aureus*** genome**.

The 16S and the metagenomic analyses were both able to match the culture inference for samples s070 and s071. The culture inference for sample s071 was MRSA and both sequencing analyses identified *S. aureus* as the top hit. However, the metagenomic analysis was able to provide additional information about a resistance profile that the 16S analysis could not. Sample s071 was the only sample for which metagenomic sequences mapped to the *mecA* gene at a significant abundance both in terms of breadth across the gene and depth in coverage (Figure 3). All 27 metagenomic sequences which mapped to the *mecA* gene during the BLAST search also map to the *S. aureus JH1* genome during the metagenomic analysis, specifically to the SaurJH1_0029 locus which corresponds to the *mecA* gene. This gene has been shown to produce a penicillin binding protein, PBP 2′, which has a low affinity for β-lactam antibiotics, including methicillin (Ubukata et al., 1989). Resistance profiles are incredibly important to clinicians and are critical for developing an appropriate antibiotic regimen. These results indicate it is possible to design sequencing based diagnostic tests which incorporate antibiotic resistance profiling along with taxonomic identification. The top hits of the 16S analysis and the metagenomic analysis of sample s070 corresponded to different species from the genus *Neisseria*. We consider this consistent with the culture inference of opportunistic flora. The top hit for the 16S analysis was *N. cinerea* and the top hit for the metagenomic analysis was *N. meningitidis*. *N. meningitidis* is found in the nasopharyngeal mucosa (Van Deuren et al., 2000) and can cause meningococcal pneumonia (Rose et al., 1981). *N. cinerea* is closely related to *N. gonorrhoeae*; while it is found in the oropharynx it has not been associated with lung infections (Knapp and Hook, 1988). The ambiguity of the culture inference does not allow for a clear distinction between the accuracy of 16S analysis and the accuracy of the metagenomic analysis. However, the metagenomic analysis does produce a top hit that is more likely to be the causative agent of pneumonia than 16S analysis top hit for sample s071.

The 16S and metagenomic analysis of samples s043 and s049 were consistent with the culture inference at the genus level, but the 16S analysis did not match the same species as the culture method. Both the metagenomic analysis and the culture approach detected the presence of *P. aeruginosa*, which is a known cause of pneumonia and often associated with nosocomial infections (Lister et al., 2009). The 16S analysis of both s043 and s049 identified *P. otitidis*, which is mainly associated with inner ear infections (Clark et al., 2006). The LTP database contains both *P. aeruginosa* and *P. otitidis*. However, the reassignment algorithm of PathoID (Francis et al., 2013) takes into account the uniqueness of the reads and for both samples there were more unique mappings to *P. otitidis* than *P. aeruginosa*. Therefore, PathoID reassigned all of the reads mapping to *P. otitidis* or *P. aeruginosa* to *P. otitidis*, resulting in the incorrect inference by the 16S analysis.

The final sample for which the 16S analysis failed to match the culture inference is sample s002. The clinical diagnosis, *Acinetobacter baumannii*, is the second hit for the 16S analysis, but is only reported as having 16.7% of the reads while the top hit, *Stenotrophomonas maltophilia*, is reported as having 83% of the reads. However, *Stenotrophomonas* is a well-known pathogen in patients who develop VAP. Whether this was the true pathogen as opposed to *Acinetobacter* cannot be determined by the data at hand.

Samples s017 and s074 were not included in the comparisons of metataxonomic and metagenomic analysis results summarized in Figure 2 because the 16S PCR failed in both samples. The metagenomic sequencing was successful and the metagenomic analysis for both samples produced a top hit of *C. glabrata*, a fungus. The metagenomic sequencing inference was confirmed, in part, by the PCRs of the fungal ITS region of both samples. The PCR products cluster with the *C. glabrata* sequences in the phylogenetic tree with a bootstrap value of 0.83 (phylogeny not shown). The culture inference for sample s017 was yeast and moderate *Klebsiella*; the metagenomic analysis has *C. glabrata* as the top hit and *K. pneumoniae* as the third hit. The culture inference of sample s074 was a moderate Gram-positive bacteria and moderate *S. pneumoniae*. The majority of the reads in s074 were *C. glabrata* and uncultured fungus, but the first bacterial identification, *S. pasteurianus*, matches the culture diagnosis at the genus level. The PCR test can only confirm the presence of *C. glabrata* in the sample and cannot confirm that *C. glabrata* is indeed the most prevalent organism. However, these results do show metagenomic sequencing is able to produce plausible results even when the 16S sequencing fails.

### The effect of sequencing on human respiratory pathogen identification

HTS, with its many advantageous characteristics, including lack of specific primer/probe design and easy multiplexing which produces large amounts of sequence data, has been instrumental in investigating the human microbiome (Turnbaugh et al., 2007). Characterization of the microbiome provides the context of a “healthy” bacterial composition to studies that investigate diseases traditionally associated with an infectious component (Beck et al., 2012) and opens the door to understanding disease as an alteration in the overall microbial community, rather than as the invasion of one particular pathogen, exemplified by the metataxonomic study of *Clostridium difficile* infections (Chang et al., 2008).

The microbiome of the lung has been particularly difficult to characterize due to prior assumptions about the community composition of the lung, the diversity of pathogens causing diseases, and sampling concerns. Until very recently, the lung was considered to be a sterile body site and it was not included as a sampling site in the original Human Microbiome Project (Beck et al., 2012). Newer studies have shown there is indeed a microbial community within the lung, but it has been difficult to characterize this community. Some studies have failed to find a distinct lung microbiome (Charlson et al., 2011), while others have found the location of the sampling within the lung will produce different results (Erb-Downward et al., 2011), or have raised concerns about the level of contamination of the lung microbiome from other body sites, such as the oropharynx (Morris et al., 2013). While the resolution is not particularly clear, there are several genera that are proposed as possible core genera of the lung microbiome including *Pseudomonas, Streptococcus, Prevotella, Fusobacterium, Haemophilus, Veillonella*, and *Porphyromonas* (Morris et al., 2013). Patients with pneumonia have been found to have microbiomes dominated by pathogens such as *S. pneumoniae, H. influenzae*, and *K. pneumoniae* (Linder et al., 2014). The core genera of the microbiome proposed by Morris et al. can be found in the metagenomic analyses. However, not all of the genera are found in all of the samples, and they are found in small proportions unless they are a top hit, such as *Pseudomonas* in samples s043 and s049. The metataxonomic analysis produced an even sparser representation of these core genera than the metagenomic analysis. Of the pathogens that are said to dominate the microbiomes of patients with pneumonia, *K. pneumoniae* is the only one found among the top hits of any sample. The results of our study have some similarities in genera with other lung microbiome studies, but these species are often found in small proportions. This may be a result of our samples coming from infected individuals, or may be because the lung microbiome is still in the process of being well-characterized.

While the results of our study were not completely congruent with other lung microbiome studies, the results did seem to correlate with respiratory pathogens. Of the top four hits of the metataxonomic and metagenomic analyses that would be considered part of the human flora or human pathogens rather than environmental pathogens, there were six organisms—*N. meningitidis* (Van Deuren et al., 2000), *Corynebacterium pseudodiphtheriticum* (Nhan et al., 2012), *K. pneumoniae* (Bratu et al., 2005), *A. baumannii* (Garnacho-Montero et al., 2003), *P. aeruginosa* (de Bentzmann et al., 1996; Lister et al., 2009), and *S. aureus* (Hooper and Smith, 2012) - that commonly cause respiratory infections. Of these respiratory pathogens, *A. baumannii, P. aeruginosa*, and *S. aureus* are commonly associated with VAP (Ashraf and Ostrosky-Zeichner, 2012). These respiratory pathogens were identified as the top hit more frequently by the metagenomic analysis than by the metataxonomic analysis. Out of the eight metagenomic analyses, five of the top hits are represented in this list of six respiratory pathogens. In the 16S analyses, only one of the six top hits fall into this category.

With only three exceptions, the top four hits in both the metataxonomic and metagenomic approaches identified microbes found in humans, either as part of the normal flora or as pathogens. These exceptions were found in both the metagenomic and the metataxonomic analyses, but the environmental microbes never accounted for more than 10% of the reads. The first exception, *Delftia acidovorans*, was identified by the metagenomic analysis in every sample except for s070 and s071. *D. acidovorans* is mainly found in soil, though it has been identified in the infection of a child with an endotracheal tube (Khan et al., 2012). The metagenomic analysis also identified *Acidovorax JS42* as the third hit for sample s002. *Acidovorax* is an environmental bacteria most often associated with plant infections, rather than human infections, though there has been one reported case of sepsis caused by *Acidovorax* (Willems et al., 1990; Shetty et al., 2005). Finally, the second hit in the 16S analysis of sample s049 was *Azomonas agilis*, an environmental bacterium that has neither been found in human flora nor been linked to an infection (Chebotar et al., 2001). These environmental microbes are not expected in the human lung. Their presence could be explained by contamination, the inhalation of soil particles by the patient or an improperly cleaned medical device, but a computational reason, such as an improperly mapped read, cannot be ruled out.

### HTS beyond primary identification of pathogens

Metagenomic analysis also presents opportunities to investigate a patient's infection that go beyond taxonomic identification of microbes. Metagenomic sequencing has been used on numerous clinical examples of antibiotic resistance pathogens to identify new genes linked to antibiotic resistance and to characterize the distribution of these genes outside the clinical setting (Forsberg et al., 2012; Wright and Poinar, 2012). All of the resulting information is being pooled in many databases, such as the Comprehensive Antibiotic Resistance Database (McArthur et al., 2013). Dual gene expression uses RNA sequences to analyze the transcriptomes of the pathogen and the host during infection (Perez-Losada et al., 2015). This should shed light on new virulence factors in the pathogens and pathways activated in response to pathogens or pathogen-associated molecular patterns as the infection progresses (Chang et al., 2011; Westermann et al., 2012). These features of metagenomics provide added value and insights into infections and potential treatment options. Metagenomics could also be useful with infections of unknown etiology due to the immense amount of genetic information captured via an unbiased method (Wilson et al., 2014) or for creating strain or population specific primers (Rohde et al., 2011).

## Conclusion

While our sample size is limited, our results indicate HTS has the potential to match standard culture techniques for the identification of bacterial pathogens. The two different HTS methods used for this study did have differences in their diagnostic capabilities and represent a tradeoff, namely between the speed of metataxonomic analyses and the accuracy and added information of metagenomic analyses. Metataxonomics is a quick, inexpensive choice when the pathogen of interest is a known bacterium, but if there are unknown pathogens, novel pathogens, a mixture of viruses, fungi and bacteria, our results show metagenomics performs better (e.g., our ability to identify fungal species with metagenomics). An effective diagnostic test must not only be fast and accurate, but must also be inexpensive, because hospitals and other healthcare facilities would be running numerous tests at anytime. Based on our results, neither HTS method can compete with the culture method in all three of these categories. However, HTS technology has seen rapid improvement over the last decade, and as these improvements continue (Shinshkin et al., 2015), HTS as a diagnostic tool will become faster and cheaper. Because diagnosis of infectious diseases is an important component of patient care, any improvements (e.g., speed, accuracy, and/or related features such as drug resistance profiles, human immune response profiles) to the diagnostic process would have an immediate impact for patients.

## Author contributions

KC, EC, IT, TM conceived of the project; IT, TM, EH, MS, GS conducted clinical work associated with the project; IT, TM collected 16S data; WJ, SH, EC, MP, KC collected metagenomic data; SH, EC, MP, IT, WJ, KC conducted data analyses; SH, EC performed fungal PCR experiments; all authors contributed to results interpretation and all contributed to reviewing and writing of the manuscript.

## Funding

SH: GW's Biology Department, Harlan Undergraduate Research Program, National Science Foundation's Research Experience for Undergraduates. EC: GW's Computational Biology Institute, “CONICYT + PAI/ CONCURSO NACIONAL APOYO AL RETORNO DE INVESTIGADORES/AS DESDE EL EXTRANJERO, CONVOCATORIA 2014 + FOLIO 82140008.” MP: K12 Career Development Program 5 K12 HL119994 award. KC, GS, MS, IT, and TM: This project was supported by Award Number UL1TR000075 from the NIH National Center for Advancing Translational Sciences. Its contents are solely the responsibility of the authors and do not necessarily represent the official views of the National Center for Advancing Translational Sciences or the National Institutes of Health. Additional support was provided by the Abramson Family Trust.

### Conflict of interest statement

The authors declare that the research was conducted in the absence of any commercial or financial relationships that could be construed as a potential conflict of interest. EC, WJ, and KC have partial ownership in a pathogen diagnostics company, Aperiomics Inc., that uses metagenomic sequencing for pathogen detection. This company provided no payment or services associated with this study.

## Footnotes

^1^Available online at: http://www.cdc.gov/nchs/ahcd.htm

## Supplementary material

Supplementary Table 1 **NCBI Accession numbers used in the phylogenetic tree and their associated species name**.

Supplementary Table 2 **Database and run statistics for the 16S and the metagenomic analysis**.

Supplementary Table 3 **Sample s043 and s049 metagenomic read proportions mapping to ***Pseudomonas aeruginosa*** strains under two different theta parameters**.
