# Supplementary material for: Metataxonomic and Metagenomic Approaches vs. Culture-Based Techniques for Clinical Pathology
Source: Front Microbiol. 2016 Apr 7;7:484. doi: 10.3389/fmicb.2016.00484 (PMC4823605; doi:10.3389/fmicb.2016.00484)
Supplement: Supplementary Table 1 — NCBI Accession numbers used in the phylogenetic tree and their associated species name. [file Table1.PDF]

Species	Tree ID	NCBI Accession Number
Malassezia globosa	Malassezia globosa	NR_111475
Candida tropicalis	Candida tropicalis [0-5]	HM771639,AF335928,HM222942, GU373664,LC042144,NR_111250
Candida rugosa	Candida rugosa [0-4]	EF568037,AF218971,AF335927, AB054033,NR_111249
Candida parapsilosis	Candida parapsilosis [0-4]	GQ497158,KF313185,KF313196, DQ317355,NR_130673
Candida glabrata	Candida glabrata [0-5]	AF218994,JN942643,AF218966, AF336836 ,AB365314,AJ293307
Candida dubliniensis	Candida dubliniensis [0-5]	EF567996,AF218993,KC408939, AF430249,AB041945,NR_119386
Candida albicans	Candida albicans [0-4]	AF335964,AF335963,GU373665, AB365317,GU373653
