# Supplementary material for: Metataxonomic and Metagenomic Approaches vs. Culture-Based Techniques for Clinical Pathology
Source: Front Microbiol. 2016 Apr 7;7:484. doi: 10.3389/fmicb.2016.00484 (PMC4823605; doi:10.3389/fmicb.2016.00484)
Supplement: Supplementary Table 2 — Database and run statistics for the 16S and the metagenomic analysis. [file Table2.PDF]

Sequencing Method	Number of reads from the sequencing run (average)	Database size (storage)	Database size (sequence count)	Average Runtime (minutes)	Average CPU time (kernel seconds)
16S	10,465	15 MB	9,672	0:07:13	50.65
Metagenomic	30,604,055	17.74 GB	7,470,039	23:01:07	80,427.72
