# Supplementary material for: Metataxonomic and Metagenomic Approaches vs. Culture-Based Techniques for Clinical Pathology
Source: Front Microbiol. 2016 Apr 7;7:484. doi: 10.3389/fmicb.2016.00484 (PMC4823605; doi:10.3389/fmicb.2016.00484)
Supplement: Supplementary Table 3 — Sample s043 and s049 metagenomic read proportions mapping to Pseudomonas aeruginosa strains under two different theta parameters. [file Table3.PDF]

Strain	S043 read proportion (default theta)	S043 read proportion (theta = 10,000)	S049 read proportion (default theta)	S049 read proportion (theta = 10,000)
Pseudomonas aeruginosa PAO1	0.76	0.58	0.80	0.78
Pseudomonas aeruginosa	0.05	0.04	0.13	0.13
Pseudomonas aeruginosa M18	N/A	N/A	0.0	0.01
Pseudomonas aeruginosa DK2	0.05	0.16	N/A	N/A
